# Developmental Paths to Anxiety in an Autism-Enriched Infant Cohort: The Role of Temperamental Reactivity and Regulation

**DOI:** 10.1007/s10803-020-04734-7

**Published:** 2020-10-09

**Authors:** Mutluhan Ersoy, Tony Charman, Greg Pasco, Ewan Carr, Mark H. Johnson, Emily J. H. Jones, The BASIS Team, Anna Blasi, Celeste Cheung, Kim Davies, Mayada Elsabbagh, Janice Fernandes, Isobel Gammer, Teodora Gliga, Jeanne Guiraud, Michelle Liew, Sarah Lloyd-Fox, Helen Maris, Louise O’Hara, Andrew Pickles, Helena Ribeiro, Erica Salamone, Leslie Tucker

**Affiliations:** 1 Department of Psychology, Institute of Psychiatry, Psychology & Neuroscience, King’s College London, 16 De Crespigny Park Denmark Hill, London, SE5 8AF UK; 2 Department of Biostatistics and Health Informatics, Institute of Psychiatry, Psychology & Neuroscience, King’s College London, London, UK; 3 Centre for Brain and Cognitive Development, Birkbeck College, University of London, London, UK; 4 Department of Psychology, University of Cambridge, Cambridge, UK

**Keywords:** Autism spectrum disorder, Anxiety, Temperament, Reactivity, Regulation, High-risk

## Abstract

The aim of this study was to explore the associations between temperamental reactivity and regulation and the emergence of anxiety traits in a longitudinal sample of infants enriched for later ASD. Parents of 143 infants who were at high- and low-risk for ASD rated their child’s temperament traits when they were 9, 15 and 24 months old; they rated anxiety and ASD traits when they were 36 months old. The findings suggest that behavioural inhibition may be an early predictor of later anxiety in children with and without ASD and that lower levels of effortful control in children who later develop ASD may contribute to the higher expression of anxiety within this population.

**Electronic supplementary material:**

The online version of this article (10.1007/s10803-020-04734-7) contains supplementary material, which is available to authorized users.

## Introduction

Diagnostic criteria for ASD encompass two core symptom domains: social communication difficulties and the presence of restricted and repetitive behaviours and sensory anomalies (American Psychiatric Association 2013). In addition to these core symptoms, a range of other mental health difficulties co-exist with ASD (e.g., Salazar et al. 2015; Simonoff et al. 2008). Anxiety is the most highly co-occurring conditions and between 40 and 70% of individuals with ASD present with at least one clinically elevated anxiety problem (Salazar et al. 2015; Simonoff et al. 2008; van Steensel et al. 2011). This rate is substantially higher than the prevalence estimate of 27% for anxiety problems in the general child population (Costello et al. 2005). At a trait level, children with ASD tended to score higher on anxiety measures compared to their typically developing (TD) peers (Bellini 2004) and children with attention deficit hyperactivity disorder (Guttmann-Steinmetz et al. 2010; van Steensel and Heeman 2017). Co-occurring anxiety and ASD symptoms may exacerbate each other and amplify the difficulties that children with ASD experience. For example, difficulties in social interaction may increase social anxiety, subsequently contributing to functional impairment (Chang et al. 2012) and reduced quality of life (van Steensel et al. 2012). Identifying the roots of anxiety in ASD may provide a basis to understand the nature of their co-occurrence, provide clear targets for early interventions, and improve long-term prognosis.

The mechanisms underlying heightened levels of anxiety in children and adolescents with ASD are poorly understood. Anxiety problems may co-occur with ASD due to common genetic factors (Hallett et al. 2013; Tick et al. 2016). Twin studies have demonstrated a tendency for increased anxiety traits in unaffected co-twins of individuals with ASD who were aged between 10 and 15 years old (Hallett et al. 2013). Cross-sectional comparisons show increased anxiety traits in unaffected infant siblings (Crea et al. 2016) and in 3 to 18 years old siblings of ASD probands (Shivers et al. 2013). Family studies indicated associations between increased parental anxiety and increased anxiety symptoms in children with ASD (Duvekot et al. 2018; Mazefsky et al. 2010). On the other hand, environmental risk factors play a crucial role in co-existence of anxiety (Mazefsky et al. 2010; van Steensel and Heeman 2017). For example, being a victim of bullying (van Schalkwyk et al. 2018), or experiencing financial challenges in the family due to the additional expense of healthcare could be important influences on childhood anxiety (Lebowitz et al. 2016). Overall, these studies suggested that there may be both genetically mediated links and environmental risks between the two disorders. However, it remains unclear whether co-occurring anxiety and ASD represent distinct disorders with independent causal pathways that happen to co-occur; whether both disorders share some common causal pathways that result in phenotypic overlap; or whether anxiety may arise as a consequence of ASD symptoms (or vice versa), an example of phenotypic causality (Wood and Gadow 2010).

In an attempt to disentangle this link, longitudinal studies have examined the bidirectional associations of anxiety and ASD in children who have received an ASD diagnosis. Duvekot et al. (2018) have shown that higher levels of anxiety traits in a group of children aged between 2 and 10 years were associated with social communication impairment over 3 years. However, Pickard et al. (2017) showed that social communication difficulties in a group of children aged between 7 and 10 years were associated with higher social anxiety symptoms measured 3 years later. The findings from these studies indicate that there are likely complex bidirectional relationships between symptoms of the two conditions once they have emerged. However, studies of children with an existing diagnosis do not provide insight into whether there are shared or distinct pathways that lead to the initial emergence of ASD and anxiety symptoms. Therefore, investigating risk factors in infancy and toddlerhood before the emergence of both disorders is crucial to understand the possible pathways for co-occurring anxiety in ASD. One promising domain of investigation is infant temperament traits since individual differences in temperament have been related to later anxiety problems in the general population (Costello et al. 2005; Nigg 2006). Thus, temperamental dispositions in infancy and toddlerhood may provide an insight into the roots of anxiety in ASD before the emergence of both disorders.

### Temperament and Anxiety in the General Population

Rothbart and Derryberry (1981) defined temperament as constitutionally based individual differences in emotional reactivity (including positive and negative affect domains) and regulation (also termed effortful control [EC]). Both reactivity and regulatory aspects of temperament play a crucial role in emotional adjustment in children (Gulley et al. 2016). Within the negative affect domain of reactivity, behavioural inhibition (BI) in infancy and toddlerhood is a robust predictor of clinically-elevated anxiety problems (e.g., Bufferd et al. 2016; Buss 2011; Scheper et al. 2017; Tang et al. 2020). BI is characterised by heightened fearfulness, shyness and wariness towards novel stimuli (Fox et al. 2001). Infants with BI may have a lower threshold and show more fearful responses to sudden changes in stimulation or inhibited approach towards novelty (Kagan et al. 1999). During toddlerhood, these children are more likely to avoid interactions with novel objects and unfamiliar people. As toddlers avoid novelty, they may become less socially competent, assertive, and subsequently be at elevated risk for anxiety disorders (Buss 2011).

The other temperamental domain that has been related to later anxiety in the general population is EC. EC emerges towards the end of the first year of life and becomes more pronounced during the preschool period (Posner and Rothbart 2000). EC refers to a child’s ability to organise reactions to external stimuli and regulate their emotions appropriately, and this can influence the severity of negative emotionality and subsequent anxiety problems (e.g., Rothbart et al. 2003). It may be that the lower levels of EC buffer the association between BI and anxiety. For example, Buss et al. (2013) found that infants who failed to regulate their emotional responses showed more BI in unfamiliar situations and more anxiety symptoms in kindergarten. In unfamiliar situations, cognitive processes (attention shifting and inhibitory control) may help children to shift their attention from threatening stimuli and inhibit extreme negative reactions that are part of BI such as sadness, fearfulness or withdrawal. Indeed, White et al. (2011) found that high levels of attention shifting (measured by an observational behavioural measure) reduced the risk of anxiety problems in children who had high levels of BI. However, in the same study high levels of inhibitory control *increased* the risk of anxiety problems. This is intriguing, since both inhibitory control and attention shifting are components of EC. Thus, whilst successfully employed EC may reduce the likelihood of heightened BI traits leading to subsequent anxiety problems, it is possible that some subcomponents of EC are less protective.

So far, evidence suggests that the combination of poor regulation and increased reactivity could represent risk factors for later anxiety symptoms in the general population. Examining whether these early temperamental capacities similarly relate to the development of anxiety in the context of familial risk for ASD design gives us an important way of testing the nature of anxiety within ASD. If anxiety is purely a downstream effect of ASD symptoms (phenotypic causality), one would not expect an identical pattern of infant temperamental predictors of anxiety to be present before behavioural symptoms of ASD have consolidated at the age of 3 when they become highly stable (Charman et al. 2005).

### Temperament and Anxiety in ASD

Most research on temperament in ASD has been conducted with children who have an existing diagnosis. In these studies, BI has not been directly studied as a specific temperamental characteristic but the broader temperament domain into which it falls (“negative affect”) and the specific BI subcomponents of shyness and fearfulness have been investigated. Broadly, children with ASD often present with reduced EC and more negative affect than typically developing peers, children with developmental delay and Fragile X across toddlerhood and childhood (e.g., Bailey et al. 2000; Brock et al. 2012; Burrows et al. 2016; De Pauw et al. 2011; Macari et al. 2017). Both higher negative affect and lower EC have been associated with elevated internalising problems in ASD (Burrows et al. 2016; De Pauw et al. 2011). However, disentangling whether these differences in temperament emerge downstream or upstream from ASD or anxiety symptoms is challenging in cross-sectional studies. We, therefore, require prospective longitudinal studies that track individuals from infancy through to early childhood when ASD symptoms fully emerge.

One fruitful research design is the study of infants with an older sibling with ASD, who have a 20% chance of developing ASD themselves (Ozonoff et al. 2011). In such studies, temperament patterns in infant siblings appear consistent with those seen in later development. Garon et al. (2016) showed that lower negative affect at 12 months was associated with higher EC at 24 months, which in turn was associated with lower levels of ASD traits at 36 months. Moreover, one previous study has provided an important initial step towards exploring what infant sibling data could tell us about the reason that anxiety and ASD often co-occur (Shephard et al. 2018). In a small group of infant siblings followed to age 7 years, increased fearfulness (a component of BI) at 14 and 24 months was correlated with both anxiety and ASD at age 7. However, in a path analysis infant fear from 7 to 24 months was associated with anxiety but not ASD symptoms when they were entered as correlated outcomes; covarying for ASD symptoms rendered the association with anxiety only marginally significant. There may be an important distinction between the component of infant fear that is stable across infancy, and developmental changes in fearful behaviour that occur over the first two years of life. One critical factor may be the interaction between infant fearfulness and EC over time; if infant fearfulness at 14 and 24 months is additionally influenced by EC over infancy and EC in infancy is related to ASD, there may be additional associations with ASD when running simple correlations at those time points that are not there when considering the component of fear that is stable from 7 months.

### Present Study

We used structural equation modelling to examine the longitudinal relationship between BI, EC, subsequent anxiety and ASD traits over the first two years of life in a group of infants with and without older siblings with ASD. BI and EC were derived from parent-reported questionnaires (the Infant Behaviour Questionnaire/IBQ in infancy and the Early Childhood Behaviour Questionnaire/ECBQ in toddlerhood). Following other studies (Crockenberg and Leerkes 2006; Gensthaler et al. 2013) BI was measured with the Fear subscale of the IBQ (which assesses a broad range of reactions to novel social and non-social stimuli) and the Shyness subscale of ECBQ (which measures wariness in social situations). EC was operationalised as the Regulatory Function subdomain of the IBQ and the Effortful Control subdomain of the ECBQ.

By including cross-lagged pathways (e.g., from a prior assessment of BI to current assessment of EC) we were able to examine how different temperament traits influence each other over developmental time and how they relate to anxiety and ASD traits. We tackled these questions in five systematic steps for parsimony and clarity of interpretation: (1) We assessed whether higher levels of BI predicts anxiety in infants at risk for ASD (Model 1) to replicate previous work by Shephard et al. (2018); specifically, we predicted that higher BI would be associated with heightened anxiety traits; (2) Since both BI and anxiety were measured through questionnaires, associations could simply reflect shared measure variance. As such, we examined specificity of the relation between BI and later anxiety by examining the relation between anxiety and a different subdomain of negative affect (i.e. sadness; Model 2). We predicted that we would not see an association between sadness and anxiety; (3) We examined how BI and EC interrelate over the first two years of life and how this associates with later anxiety (Model 3). Specifically, we predicted that a combination of lower levels of EC and higher levels of BI would predict later anxiety traits; (4) We examined how BI and EC interrelate over the first two years of life and how this associates with later anxiety and ASD (Model 4). Specifically, we predicted that there would be associations between lower EC, greater BI and more symptoms of both anxiety and ASD. (5) We used mediation analysis to probe the relationship between BI, EC, and later anxiety/ASD over time. Mediation analysis allowed us to ask whether the relationship between an infant predictor and a developmental outcome can be fully accounted by another variable; this will enable us to address whether the association between temperament traits and anxiety/ASD is exacerbated by the co-occurring conditions. We predicted that anxiety would mediate any relationship between BI and ASD symptoms but that EC would be more generally related to lower risk for both anxiety and ASD, given proposals of its role as a general protective factor (Johnson 2012).

## Method

### Participants

Participants in this study were 116 high-risk (HR) (52 female; 64 male) and 27 low-risk (LR) (13 female; 14 male) children who took part in the longitudinal British Autism Study of Infant Siblings (BASIS; www.basisnetwork.org). All HR participants had at least one older sibling with a community clinical diagnosis of ASD (herein, proband). Diagnosis of probands was confirmed by expert clinicians (TC, PB) based on parent-reported Development and Wellbeing Assessment (DAWBA; Goodman et al. 2000), the Social Communication Questionnaire (SCQ; Rutter et al. 2003), or parent-confirmed community clinical ASD diagnosis (see Supplementary Material Section 1 for further details).

The LR group had at least one TD older sibling and no first-degree family members with an ASD diagnosis (confirmed through family medical history screening). LR infants were recruited from a volunteer database at the Birkbeck Centre for Brain and Cognitive Development. Possible ASD symptoms were screened in the older siblings of the LR infants with the SCQ and none of the older siblings scored above the ASD cut-off score of 15. Further inclusion criteria for both groups were having at least one parent speaking English at home.

Exclusion criteria for both groups, based on parent report, included significant prematurity (gestational age ≤ 32 weeks; groups did not differ based on the gestational age *χ*^*2*^ (3) = 3.18, *p* = 0.364), marked medical conditions such as epilepsy, heart conditions, vision and hearing impairments, cerebral palsy, or genetic conditions such as Down’s syndrome or Fragile X, parents with evidence of learning difficulties or unable to give informed consent. No infants had any known medical or developmental condition at the time of enrolment.

Families enrolled in this study when their babies were 8 months of age or younger and attended four lab visits when children were 9, 15, 24 and 36 months of age. A battery of questionnaires was posted to parents before each visit and parents were asked to bring them to the laboratory visit.

Ethical approval for this study was given by the National Health Service National Research Ethics Service (NHS NRES London REC 08/H0718/76). At each time point, written informed consent was obtained from parents for their children to participate in the study.

Clinical outcome decision for the current cohort was summarised in the Supplementary Material Section 1.

### Measures Used in Modelling

#### Temperament

Fear, shyness, sadness and EC were measured using the Infant Behaviour Questionnaire-Revised (IBQ-R; Gartstein and Rothbart 2003) at 9 and 15 months, and the Early Childhood Behaviour Questionnaire (ECBQ; Putnam et al. 2006) at 24 months. These are parent-report questionnaires that allow caregivers to rate the frequency of particular behaviours during the previous 2 weeks. The factor analysis of both questionnaires yielded three higher-order factors: (i) Surgency and (ii) Negative Affect, which constitute the reactivity domain, and (iii) EC, which constitutes the self-regulatory domain. In both measures, sadness refers to general low mood and activity related to personal suffering, physical state, object loss, or inability to perform a desired action and EC characterised by the ability of shifting attention, duration of attentional focusing, inhibitory control and low-intensity pleasure.

BI was measured using the Fear subscale of the IBQ-R (at 9 and 15 months), and the Shyness subscale of the ECBQ (at 24 months). In the IBQ-R, the Fear subscale measures infant distress or inhibited approach to both novel social and non-social stimuli. Initial development work on the IBQ-R indicated that the social and non-social components could not be reliably dissociated in infancy (Gartstein and Rothbart 2003). However, this is not the case in toddlerhood and so in the ECBQ this collection of behaviours is separated into two subscales termed Fear (covering distress or inhabited approach to novel non-social stimuli) and Shyness (referring to discomfort, slow or inhibited approach to novelty and uncertainty in social situations). In the current sample, the longitudinal correlation between the IBQ-Fear at 15 months and ECBQ-Fear at 24 months was *r* = 0.40, *p* = 0.001; the correlation between the IBQ Fear at 15 months and the ECBQ-Shyness at 24 months was *r* = 0.52, *p* < 0.001. To remain faithful to the factor structure of the ECBQ, we thus selected the Shyness subscale at 24 months because of its closer association to IBQ-Fear. This approach is also supported by past studies, which showed significant cross-sectional and longitudinal associations between Fear and Shyness subscales in infancy and toddlerhood (Eggum et al. 2009; Wolfe et al. 2014). However, to assess the robustness of this approach we additionally repeated analyses with a composite of the ECBQ Fear and Shyness subscales (see Supplementary Material Section 7); the pattern of results was the same.

#### Anxiety Traits

Anxiety traits were measured with the DSM-Oriented Anxiety Problems subscale of the Child Behaviour Checklist 1.5/5 at 36 months (CBCL; Achenbach and Rescorla 2000). The CBCL is a standardised parent-report questionnaire measuring emotional, behavioural and social problems. Parents endorse one of the item responses 0 ‘Not true,’ 1 ‘Somewhat or Sometimes True,’ or 2 ‘Very True or Often True’ to specify the frequency of problems that the child has experienced in the past 2 months.

#### ASD Traits

ASD symptomatology was measured using the Social Communication Questionnaire (SCQ; Rutter et al. 2003) at 36 months. The SCQ is a 40-item parent-reported questionnaire which allows parents to endorse ASD related behaviours that they have observed in their children over the past three months. Total SCQ scores vary between 0 and 39 and higher scores reflect higher symptoms.

### Data Analysis

Pearson correlation coefficients were calculated to assess the relationships between predictors (temperament traits measured at 9, 15, and 24 months) and outcomes (anxiety and ASD measured at 36 months) for the HR and LR groups separately (Table 2). Due to the high number of comparisons made, the reported significance level was set to *p* < 0.001. Mean group differences, between LR and HR participants, were computed for all predictors and outcome variables using SPSS 23.

Cross-Lagged and mediation models were estimated in a structural equation modelling (SEM) framework using Mplus 7.13 (Muthén and Muthén 1998–2015). Full Information Maximum Likelihood (FIML) was used to account for missing data and Maximum Likelihood Robust (MLR) estimation was used to provide robust standard errors to account for the non-normal distributions and skewness in the anxiety outcome measure. Model fit was assessed by the root means square error of approximation (RMSEA), comparative fit index (CFI) and standardised root mean square residual (SRMR). An acceptable fit was indicated by RMSEA of 0.05–0.08, CFI of 0.90–0.95, SRMR of 0.05 − 0.08. A good fit was indicated by RMSEA of 0.01–0.05, CFI of 1–0.95, and SRMR of < 0.05 (Hu and Bentler 1999; Kline 2016). All model coefficients are standardised with respect to the predictor and outcome (i.e. STDYX in Mplus; Muthén and Muthén 1998–2015).

We fit models sequentially for parsimony and clarity of interpretation. All models were estimated using observed (i.e. non-latent) variables only. In the main text, we reported the models that were estimated using only the HR sample (*N* = 116). To maximise power, we then estimated the same models using combined the HR and LR groups (*N* = 143) and summarised in the Supplementary Material Section 3. In these models, the risk-groups (LR = 0; HR = 1) was treated as a covariate in all models to control for effect and regressed on each predictor and outcome variables. Of note, we present the Pearson correlation coefficients summarised for the HR and LR groups combined in the Supplementary Material Section 2.

**Fig. 1:**
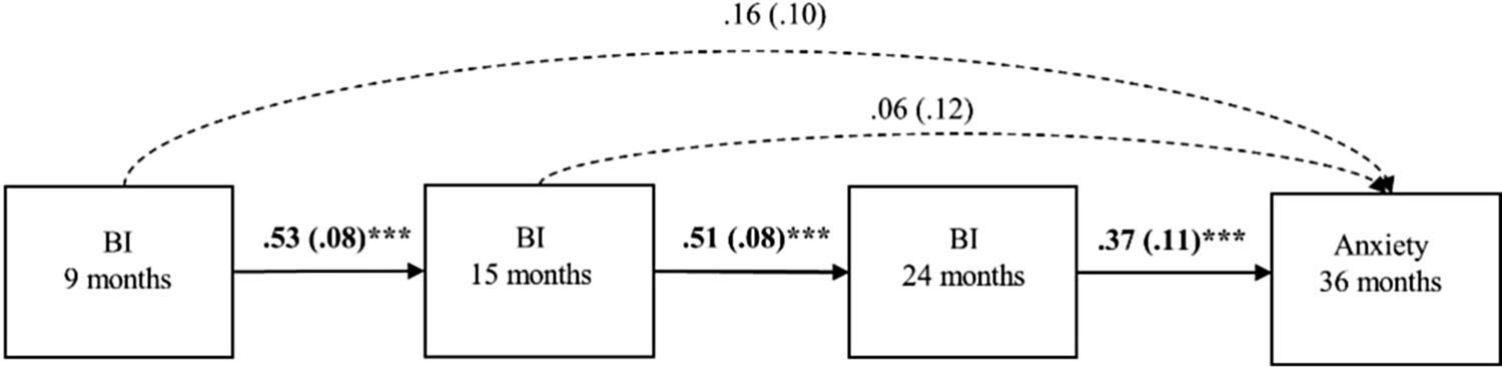
Model 1, the first-order autoregressive model of behavioural inhibition and anxiety. Bolds indicate significant association, standardised beta and standard errors are reported. ***p < 0.001, **p < 0.01, *p < 0.05

As a baseline model, we tested the relationship between infant BI (9, 15, 24 months) and anxiety in toddlerhood (36 months) in Model 1, illustrated in Fig. 1. This model included autoregressive pathways between three measures of BI at 9, 15, and 24 months. It further included direct pathways between each BI measure and anxiety traits at 36 months, to assess the developmental timing of the association.

**Fig. 2:**
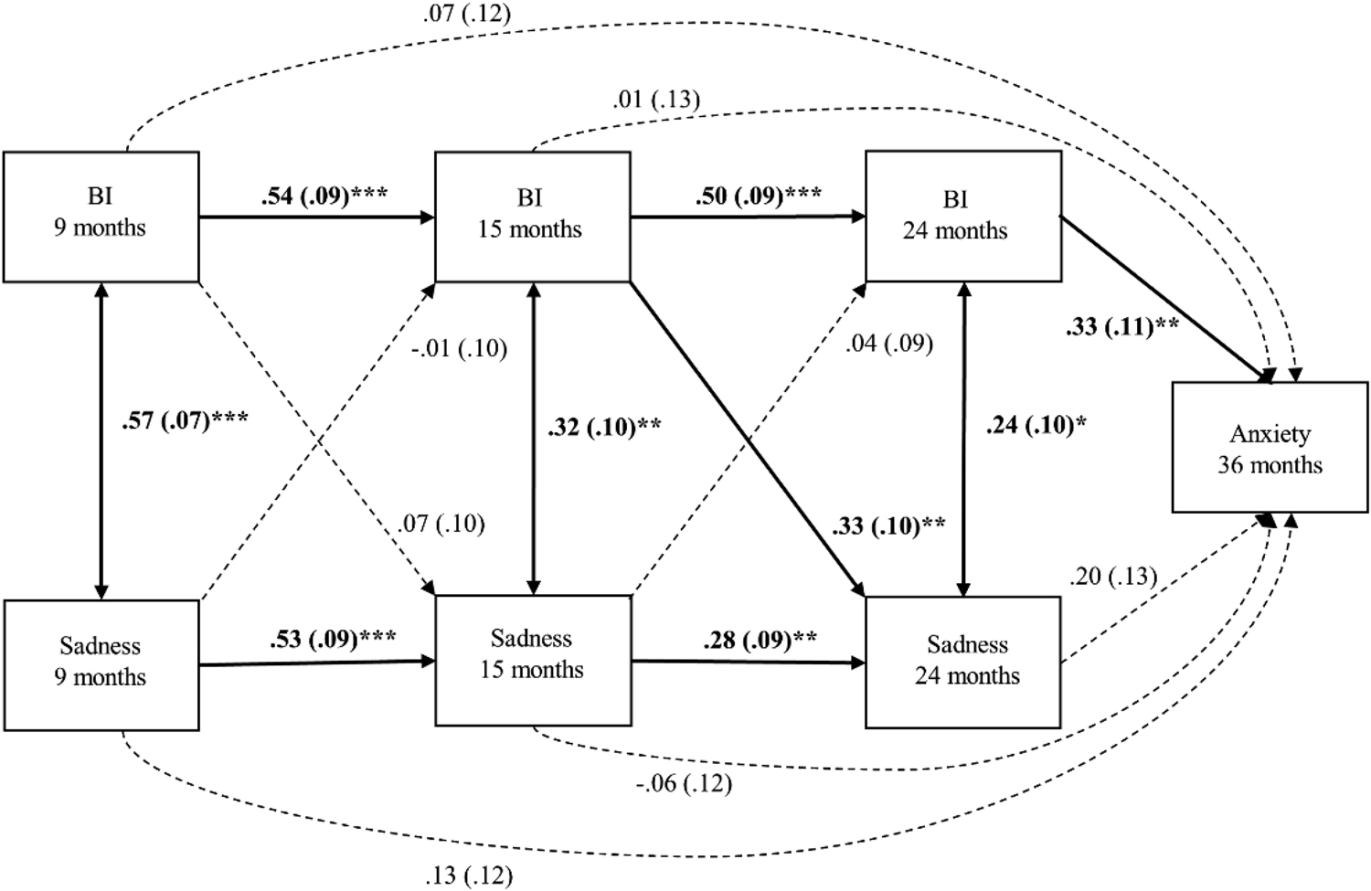
Model 2, cross-lagged association between behavioural inhibition, sadness and anxiety. Bolds indicate significant association, standardised beta and standard errors are reported. ***p < 0.001, **p < 0.01, *p < 0.05

In Model 2, we tested the specificity of BI by using a cross-lagged model to measure longitudinal and concurrent relationships between BI and sadness, and their association with anxiety traits at 36 months (Fig. 2).

**Fig. 3:**
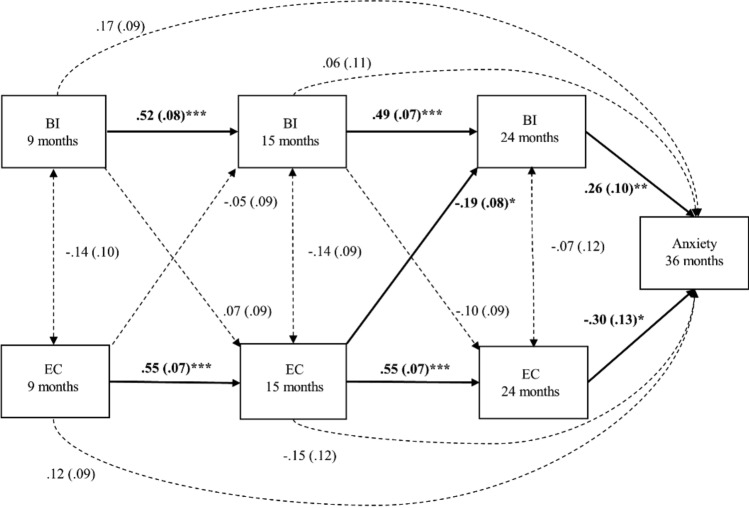
Model 3, cross-lagged association between behavioural inhibition, effortful control and anxiety. Bolds indicate significant association, standardised beta and standard errors are reported. ***p < 0.001, **p < 0.01, *p < 0.05

We used a cross-lagged model to examine the direction of longitudinal relationships between these EC, BI and later anxiety in Model 3. Direct pathways between each temperament traits and anxiety traits were incorporated in this model (Fig. 3).

**Fig. 4:**
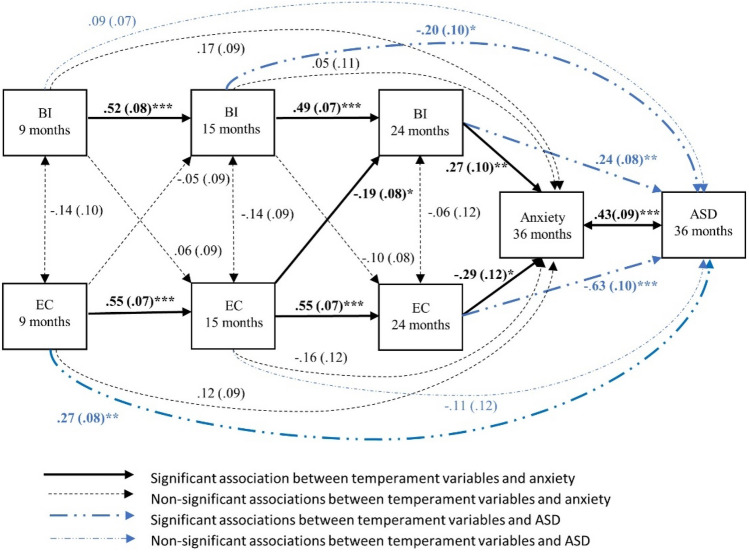
Model 4, cross-lagged association between behavioural inhibition, effortful control, anxiety and asd. Standardised beta and standard errors are reported. ***p < 0.001, **p < 0.01, *p < 0.05

In Model 4, we extended Model 3 by including ASD scores as a secondary outcome variable to assess whether the findings in the Model 3 are unique to anxiety or also shared with ASD (Fig. 4).

### Exploratory Mediation Analysis

To explore whether the relationships between BI, EC (24 months) and anxiety (36 months) were mediated by ASD (36 months), or conversely, whether the association between BI and EC (24 months) and ASD (36 months) were mediated by anxiety (36 months), two separate mediation models were estimated. For clarity, these models included the exposure, mediator, and outcomes only and did not incorporate the cross-lagged panel structure used above. In both mediation models, indirect effects were estimated using Maximum Likelihood estimation with bootstrapped confidence intervals (1000 iterations).

## Results

### Sample Characteristics

Sample characteristics, means and standard deviations for measures and risk group comparisons (effect sizes) are shown in Table 1. Groups did not differ in the proportion of girls and were the same age at each visit with the exception of the 24-month timepoint where the HR group were older than the LR controls. The HR group scored significantly higher than the LR group on the BI subscale at 9 and 15 months but not at 24 months. The HR group had significantly lower EC than the LR group only at 24 months. The HR group scored higher than the LR group on the anxiety subscale and ASD total scores at 36 months.

**Table 1:** Sample characteristics and descriptives by risk group

	High-risk M (SD)	Low-risk M (SD)	Group differences
Visit 1–9 months			
N (girls)	116 (52)	27 (13)	*χ*^*2*^ (1) = 0.002, *p* = 0.960
Age in days	274.18 (24.81)	283.42 (25.36)	*t* (138) = 1.71, *p* = 0.090, *d* = 0.37
IBQ BI	3.03 (1.13)	2.60 (0.76)	*t* (52.99) = − 2.37, *p* = 0.021, *d* = 0.45
IBQ sadness	3.86 (0.95)	3.69 (0.97)	*t* (138) = − 0.83, *p* = 0.407, *d* = 0.18
IBQ effortful control	4.68 (0.65)	4.75 (0.69)	*t* (138) = 0.456, *p* = 0.649, *d* = 0.10
Visit 2–15 months			
N (girls)	113(51)	27(13)	*χ*^*2*^ (1) = 0.102, *p* = 0.749
Age in days	465.90 (30.36)	473.63 (27.82)	*t* (139) = 0.83, *p* = 0.409, *d* = 0.27
IBQ BI	3.47 (1.05)	2.94 (0.88)	*t* (135) = − 2.41, *p* = 0.017, *d* = 0.55
IBQ sadness	3.99 (0.97)	3.76 (0.83)	*t* (136) = − 1.17, *p* = 0.244, *d* = 0.25
IBQ effortful control	4.57 (0.63)	4.83 (0.60)	*t* (136) = 1.98, *p* = 0.050, *d* = 0.42
Visit 3–24 months			
N (girls)	102(46)	27 (13)	*χ*^*2*^ (1) = 0.01, *p* = 0.916
Age in months	26.26 (2.23)	24.71 (1.20)	*t* (120) = − 3.28, *p* = 0.001, *d* = 0.87
ECBQ BI	3.25 (1.07)	3.06 (0.88)	*t* (120) = − 0.82, *p* = 0.417, *d* = 0.19
ECBQ sadness	3.11 (1.00)	2.55 (0.60)	*t* (58.69) = − 3.53, *p* = 0.001, *d* = 0.68
ECBQ effortful control	4.45 (0.72)	4.72 (0.41)	*t* (61.79) = 2.45, *p* = 0.017, *d* = 0.46)
Visit 4–36 months			
N (girls)	111 (49)	25 (11)	*χ*^*2*^ (1) = 0.001, *p* = 0.983
Age in months	38.88 (1.96)	38.72 (1.46)	*t* (127) = − 0.37, *p* = 0.712, *d* = 0.09
MSEL ELC	102.46 (25.10)	119.48 (15.26)	*t* (57.49) = 4.40, *p* < 0.001, *d* = 0.82
CBCL anxiety problems	3.22 (3.42)	1.92 (1.53)	*t* (82.19) = − 2.75, *p* = 0.008, *d* = 0.49
SCQ	6.26 (6.84)	2.64 (2.29)	*t* (115.23) = − 4.52, *p* < 0.001, *d* = 0.71

*MSEL ELC* mullen scales of early learning early learning composite standard score, *IBQ* infant behaviour questionnaire, *ECBQ* early childhood behaviour questionnaire, *CBCL* child behaviour checklist, *SCQ* social communication questionnaire total scores

We calculated correlation coefficients separately for the HR and LR groups. The results indicated that the associations between temperament variables at three timepoints, temperament and later anxiety and ASD traits were not significant at the *p* < 0.001 level in the LR group. In the HR group, the associations between BI at 15 and at 24 months, Sadness at 24 months and EC at 24 months were significant at *p* < 0.001 level (*r* =  − 0.35, *r* = 0.44, *r* = 0.403, *r* =  − 0.43; respectively); the associations between EC at 15 and 24 months, anxiety at 36 months and ASD were significant at *p* < 0.001 level (*r* =  − 0.35, *r* =  − 0.61, *r* = 0.57; respectively). As shown in Table 2, the HR group showed a relatively comparable pattern to the cohort as a whole and there were enough variations between predictors and outcomes for running all the hypothesised models in the HR group.

**Table 2:** Pearson correlation coefficients of research variables split by the groups, above the diagonal represent the LR group (N = 27) and below the diagonal represents the HR group (N = 116)

	1	2	3	4	5	6	7	8	9	10	11	12
1. IBQ fear 9 m	–	0.576	0.386	0.152	0.444	0.368	0.505	0.072	− 0.046	− 0.230	0.174	0.333
2. IBQ fear 15 m	0.535*	–	0.466	0.032	0.227	0.255	0.409	0.247	0.102	− 0.269	0.378	0.566
3. ECBQ shyness 24 m	0.283	0.526*	–	0.658*	0.560	0.277	0.331	0.041	− 0.162	− 0.225	0.379	0.192
4. ECBQ fear 24 m	0.447*	0.480*	0.577*	–	0.423	0.210	0.134	− 0.069	− 0.080	− 0.049	0.170	0.045
5. IBQ sadness 9 m	0.567*	0.291	0.107	0.245	–	0.539	0.591	− 0.124	− 0.021	− 0.342	0.047	0.047
6. IBQ sadness 15 m	0.373*	0.405*	0.259	0.339	0.560*	–	0.520	0.091	− 0.232	− 0.147	− 0.070	0.127
7. ECBQ sadness 24 m	0.359*	0.452*	0.424*	0.442*	0.455*	0.424*	–	0.043	0.213	− 0.193	− 0.186	0.085
8. IBQ effortful control 9 m	− 0.136	− 0.121	− 0.189	-0.117	− 0.088	− 0.101	− 0.116	–	0.630	0.513	− 0.042	0.284
9. IBQ effortful control 15 m	− 0.021	− 0.133	− 0.247	− 0.183*	0.017	− 0.089	− 0.127	0.543*	–	0.266	− 0.398	0.236
10. ECBQ effortful control 24 m	− 0.072	− 0.172	− 0.230	− 0.195	− 0.194	− 0.110	− 0.221	0.429*	0.549*	–	− 0.091	0.019
11. CBCL anxiety problems 36 m	0.301	0.352*	0.444*	0.404*	0.275	0.240	0.403*	− 0.151	− 0.338	− 0.428*	–	0.456
12. SCQ 36 m	0.074	0.076	0.318	0.246	0.129	0.070	0.257	− 0.065	− 0.353*	− 0.608*	0.574*	–

IBQ infant behaviour questionnaire, *ECBQ* early childhood behaviour questionnaire, *CBCL* child behaviour checklist, *SCQ* social communication questionnaire

^*^p < 0.001

### Model 1: Longitudinal Association between BI and Anxiety

We first tested the hypothesised relationship between infant BI related and toddler anxiety (Fig. 1). Overall, we found association between BI (24 months) and anxiety traits (36 months). The fit indices of the auto-regressive model indicated a good fit to the data (*χ*^*2*^ (1) = 0.094, *p* = 0.759; CFI = 1.00, RMSEA = 0.000, and SRMR = 0.006). All coefficients for the autoregressive pathways of BI were significant; BI at 9 months was related to subsequent BI at 15 months (*β* = 0.53, *p* < 0.001) and BI at 15 months associated with BI scores at 24 months (*β* = 0.51, *p* < 0.001), with the similarity in standardised beta values indicating stability in magnitudes of associations between time points. Higher levels of BI at 24 months were associated with higher levels of anxiety at 36 months (*β* = 0.37, *p* < 0.001).

### Model 2: Specificity of BI in Predicting Anxiety

Overall model fit for the second model was acceptable (CFI = 0.948, RMSEA = 0.137, and SRMR = 0.040), despite significance for the model (*χ*^*2*^ (4) = 12.65, *p* = 0.013; Fig. 2). The autoregressive pathways indicated a significant association in BI between 9 and 15 months (*β* = 0.54, *p* < 0.001); and 15 and 24 months (*β* = 0.50, *p* < 0.001). There was also significant association in sadness between 9 and 15 months (*β* = 0.53, *p* < 0.001) as well as 15 and 24 months (*β* = 0.28, *p* = 0.003). BI and sadness was associated with each other concurrently at each time point (9 months: *β* = 0.57, *p* < 0.001; 15 months: *β* = 0.32, *p* = 0.001; 24 months: *β* = 0.24, *p* = 0.017) but the magnitude of the relationship decreased over time.

Cross-lagged paths indicated that more BI at 15 months is related to more sadness at 24 months (*β* = 0.33, *p* = 0.001) and the rest of the cross-lagged associations were not significant (all *p* ≥ 0.480). Similar to the Model 1, greater BI at 24 months was significantly related to higher anxiety scores at 36 months (*β* = 0.33, *p* = 0.002; Model 2) but there were no significant relationships between sadness and anxiety problems at any time point (all *p* ≥ 0.120). Thus, the predictive relation between BI and anxiety was not shared with sadness and BI was used in the rest of the analysis as a subdimension of negative reactivity.

### Model 3: Longitudinal Association between BI, EC and Anxiety

We then tested how infant EC interrelated with infant BI in the prediction of later anxiety (Fig. 3). The cross-lagged auto-regressive model provides a good fit to the data (*χ*^*2*^ (4) = 3.07, *p* = 0.547; CFI = 1.00, RMSEA = 0.000, and SRMR = 0.024). The autoregressive pathways indicate that BI (9–15 months: *β* = 0.52, *p* < 0.001; 15–24 months: *β* = 0.49, *p* < 0.001) and EC (9–15 months: *β* = 0.55, *p* < 0.001; 15–24 months: *β* = 0.55, *p* < 0.001) had a stable magnitude of relationship between different timepoints. Concurrent residual correlations between BI and EC were not significant (*p* ≥ 0.140), which means that BI and EC were not interrelated within a timepoint. As for the cross-lagged paths, higher levels of EC at 15 months related to decreased BI at 24 months (*β* =  − 0.19, *p* = 0.017). Both higher levels of BI and lower levels of EC at 24 months were significantly associated with higher levels of anxiety symptoms (*β* = 0.26, *p* = 0.008; *β* =  − 0.30, *p* = 0.017; respectively). Thus, better EC at 15 months appeared to be associated with lower anxiety through decreased BI in toddlerhood.

### Model 4: Longitudinal Association between BI, EC, Anxiety and ASD

We then tested how ASD traits interrelate with infant temperament and later anxiety (Fig. 4). The model fit indices suggested a good fit to the data (*χ*^*2*^ (4) = 2.16, *p* = 0.707; CFI = 1.00, RMSEA = 0.000, and SRMR = 0.020). The magnitude of the autoregressive pathways for BI and EC were stable from infancy to toddlerhood (BI: 9–15 months: *β* = 0.52, *p* < 0.001; 15–24 months: *β* = 0.49, *p* < 0.001. EC: 9–15 months: *β* = 0.55, *p* < 0.001; 15–24 months: *β* = 0.55, *p* < 0.001). Concurrent correlations between BI and EC were not significant at all three timepoints (*p* ≥ 0.139).

As in the previous model, the cross-lagged paths indicated that higher EC at 15 months related to decreased BI at 24 months (*β* =  − 0.19, *p* = 0.017). Both higher levels of BI and lower levels of EC at 24 months were significantly associated with heightened levels of anxiety symptoms (*β* = 0.27, *p* = 0.007; *β* =  − 0.29, *p* = 0.016; respectively) and ASD symptoms (*β* = 0.24, *p* = 0.002; *β* =  − 0.63, *p* < 0.001; respectively). Unlike for anxiety, lower levels of BI at 15 months was inversely related to ASD traits (*β* =  − 0.20, *p* = 0.036) and higher levels of EC at 9 months were related to increased ASD symptoms (*β* = 0.27, *p* = 0.001). There was also a concurrent positive relationship between anxiety and ASD symptoms at 36 months (*β* = 0.43, *p* < 0.001). Thus, this model indicates that both BI and EC are associated with anxiety and ASD symptoms in toddlerhood, but there may be additional specific relations between EC in early infancy and later ASD.

### Exploratory Mediation Analysis

We examined whether toddler BI and EC were equally related to anxiety and ASD symptoms. There was evidence of an indirect effect of BI (24 months) on anxiety (36 months) via ASD (36 months; *β* = 0.15, *p* = 0.003, 95% CI [0.08, 0.25]). The direct (*β* = 0.29, SE = 0.09, *p* = 0.001), and the total effects *(β* = 0.44, SE = 0.11, *p* < 0.001) were significant, suggesting a partial mediation. Specifically, 34% of the total effect of BI on anxiety was operating through ASD. There was also a significant indirect effect of BI on ASD through anxiety (*β* = 0.24, *p* = 0.001, 95% CI [0.13, 0.36]). The direct effect of BI on ASD was not significant *(β* = 0.07, SE = 0.09, *p* = 0.448), and the total effect was significant *(β* = 0.31, SE = 0.10, *p* = 0.003), anxiety mediated (complete mediation) the relationship between BI and ASD and accounted for 79% of the total effect. Thus, these results suggest that BI is more strongly related to anxiety rather than ASD traits.

The indirect effect of EC (24 months) on anxiety (36 months) via ASD (36 months) was significant (*β* =  *− *0.33, *p* < 0.001, 95% CI [− 0.43, − 0.22]). The direct effect was not significant (*β* =  − 0.09, SE = 0.10, *p* = 0.355), and the total effect was significant *(β* =  − 0.42, SE = 0.08, *p* < 0.001), suggesting a complete mediation. Seventy-nine per cent of the total effect of EC on anxiety was operating through ASD. There was also a significant indirect effect of EC on ASD through anxiety (*β* =  − 0.16, *p* = 0.003, 95% CI [− 0.27, − 0.09]). The direct effect *(β* =  − 0.46, SE = 0.09, *p* < 0.001), and the total effect were both significant *(β* =  − 0.62, SE = 0.07, *p* < 0.01). Anxiety partially mediated the association between EC and ASD, accounting for the 26% of the total effect (Table 3). Thus, the pattern for EC was the opposite as for BI, suggesting that EC at 24 months is more strongly related to ASD than anxiety.

**Table 3:** Summary of exploratory mediation analyses

Predictor	Mediator	Outcome	Total effect	Direct effect	Indirect effect (95% CI Bootstrap)	Percentage of total effect mediated
1) BI 24 m	ASD 36 m	Anxiety 36	0.44 (0.11) ***	0.29 (0.09) **	0.15 (0.08, 0.25) **	34
2) Effortful control 24 m	ASD 36 m	Anxiety 36	− 0.42 (0.08) ***	− 0.09 (0.10)	− 0.33 (-0.43, − 0.22) ***	79
3) BI 24 m	Anxiety 36 m	ASD 36	0.31 (0.10) **	0.07 (0.09)	0.24 (0.13, 0.36) **	77
4) Effortful control 24 m	Anxiety 36 m	ASD 36	− 0.62 (0.07) ***	− 0.46 (0.09) ***	− 0.16 (− 0.27, − 0.09) **	26

^***^p < 0.001, **p < 0.01, *p < 0.05

### Sensitivity Analyses

All models were re-estimated to control for the effect of possible confounders sex and cognitive ability. The effect of missing data was addressed by re-estimating the models with listwise deletion. Pearson correlation coefficients were carried out on the HR and the LR groups combined and all models were re-run in the combined HR and LR groups to address the power issue. The results of the models after adjustment for sex and with listwise deletion did not change. However, when running all analyses in the combined HR and LR groups, the association between 15 month BI and the 36 months ASD traits were not significant; when controlling for cognitive ability at 36 months the relationship between BI at 9 months and anxiety at 36 months, BI at 15 months and ASD at 36 months become significant and the association between 15 months EC and 24 months BI was not significant (*p* = 0.06) and association between BI, EC at 24 months and outcome variables at 36 months remains significant; all other patterns of findings remain the same. Further results for models 1 to 4 are summarised in the Supplementary Material Section 2–7.

## Discussion

The primary aim of this prospective study was to examine the developmental pathways that contribute to the co-occurrence between anxiety and ASD traits. We investigated how infant reactivity and regulation related to later anxiety in a cohort enriched for later ASD symptoms. The models showed that (1) higher levels of BI at 24 months were associated with anxiety traits at 36 months and this association was not shared with other aspects of infant negativity; (2) more BI at 24 months was associated with more anxiety traits at 36 months, although this was reduced by having better EC at 15 months. (3) there was a similar association between reduced EC, higher BI and ASD; (4) mediation analyses showed that BI was related to ASD traits through emerging anxiety traits whereas EC was related to anxiety through ASD traits, which indicates separate developmental pathways for anxiety and ASD.

### Infant BI and Anxiety

Model 1 confirmed that heightened reactivity in infancy was associated with higher levels of anxiety in our cohort, consistent with the existing literature in the general population (Bufferd et al. 2016; Buss 2011). Specifically, heightened BI at 24 months was related to heightened levels of anxiety scores at 36 months. This is consistent with a previous study showing that shyness in toddlerhood was significantly correlated with anxiety symptoms in a cohort of infant siblings followed up to age 7 (Shephard et al. 2018).

Prior evidence has suggested increased negative affect (Macari et al. 2017) and BI in children with an ASD diagnosis (Brock et al. 2012), and temperament traits have been investigated in relation to ASD traits at 36 months in infants at risk of ASD (Garon et al. 2016). However, few studies have examined associations between these temperament traits and emerging internalising problems (though see Burrows et al. 2016; De Pauw et al. 2011; Shephard et al. 2018). Thus, Model 2 assessed whether the observed associations between BI and anxiety in the present sample were specific, or whether they may extend to other aspects of negative affect, specifically sadness. Planalp et al. (2017) argued that BI and sadness share similar reactions such as withdrawal, but the context in which we observe them differs. Specifically, BI is characterised by reactions to novelty or over-arousing stimuli whereas sadness is characterised by broader reactions to goal blockage or loss without an approach/withdrawal orientation. Our results showed a significant concurrent association between BI and sadness at each time point and higher BI in infancy was related to increased sadness in toddlerhood (Model 2). However, infant/toddler sadness was not related to anxiety outcome. In line with Planalp et al. (2017) concurrent association at each time point may indicate that the association between BI and sadness share similar underlying features (e.g., withdrawal) in the current cohort but the relationship between infant BI and anxiety confirms that prediction of anxiety is specific to BI.

Following Planap et al. (2017), infants’ intolerance of uncertainty in a novel context may explain why infants’ anxiety is specifically related to BI. Intolerance to uncertainty is the perception that an uncertain, unpredictable context is stressful and threatening, and this perception results in avoidance (Anderson et al. 2012). Indeed, in children and young adults with ASD, intolerance to uncertainty may be the mechanism that explains the variation between anxiety and ASD traits (Boulter et al. 2014). Thus, elevated worry in ambiguous or over-arousing situations may be an early form of intolerance to uncertainty, which results in maladaptive emotionality in infant siblings.

### Longitudinal Association Between BI, EC and Anxiety

Model 3 shows how EC interrelates with BI in the prediction of later anxiety. EC has been proposed to be a protective factor against a wide range of conditions including both ASD (Johnson 2012) and anxiety (White et al. 2011). Thus, EC would be expected to interrelate with other risk factors in shaping later psychopathology. The cross-lagged approach we employed allowed us to test this possibility directly. Specifically, we explored the longitudinal directionality of the association between BI and EC over the time window in which behavioural symptoms of ASD and anxiety emerge. As expected, better EC at 15 months was associated with less BI at 24 months. Furthermore, successfully employed EC in infancy reduced the levels of dysregulated BI in toddlerhood, in turn, lower levels of BI in toddlerhood reduced the likelihood of presenting anxiety traits at 36 months. This is consistent with the proposal that EC and related constructs may be broad protective factors against later psychopathology (Johnson 2012).

### Longitudinal Association between BI, EC, Anxiety and ASD

Since symptoms of anxiety and ASD intertwine, it is also crucial to further investigate whether infant predictors related similarly or differently to ASD and anxiety outcomes. Model 4 showed that for some paths, BI and EC related similarly to later anxiety and ASD traits. Specifically, higher EC at 15 months related to lower levels of BI at 24 months; and lower levels of BI at 24 months related to both fewer anxiety and ASD traits (36 months). This may suggest shared developmental pathways to both anxiety and ASD traits. Alternatively, infant temperamental features may have apparently similar relations to both ASD and anxiety because phenotypic causality drives stronger associations between ASD and anxiety symptoms once they have emerged. To dissect this possibility, we used mediation analyses to explore whether BI and EC at 24 months relate to ASD traits through co-occurring anxiety symptoms, or whether BI and EC relate to anxiety symptoms through ASD traits. This could tell us, for example, whether more fearful infants develop anxiety symptoms that then exacerbate their ASD symptoms, or whether infants who have poorer EC develop ASD symptoms that then lead to anxiety symptoms.

Our results suggest differences between EC and BI in terms of their relationship with ASD and anxiety traits. We found that early BI predicted later anxiety symptoms, and this was only partially mediated by ASD symptoms. In contrast, the relation between early BI and later ASD symptoms were fully mediated by anxiety. This means that the degree to which early reactivity predicts later ASD is likely attributable to more anxious children tending to have higher levels of ASD symptoms in our cohort. This association could be due to phenotypic causality such that anxiety exacerbates social difficulties (Duvekot et al. 2018). These results suggest that apparent infant predictors of later ASD in sibling studies are actually related to risk for co-occurring conditions like anxiety or ADHD.

Our results diverge from those observed in a slightly smaller cohort in which Shephard et al. (2018) showed that shyness at 24 months was related to 7-year ASD but not anxiety symptoms. Our observation of associations between 24 months BI and anxiety may have been due to more power associated with our larger sample (*N* = 143 in our study, compared to *N* = 104 in Shephard et al. (2018)). Further, our statistical approach examined the longitudinal bidirectional effects between BI and EC (see also, Eggum et al. 2009; Wolfe et al. 2014), whereas Shephard et al. (2018) specifically tested the relation between shyness at 24 months and anxiety scores at 7 years. Incorporating longitudinal data and taking into account the bidirectional relation between EC and BI may reveal patterns of association that are not detectable when focusing on simple associations. Alternatively, there may be stronger associations between toddler BI and early-emerging anxiety traits at age 3 than in later development, where other cascading effects and phenotypic interactions further complicate the relationship. Some studies suggest that anxiety symptoms increase from childhood to young adulthood (Gotham et al. 2015; van Steensel et al. 2011), less is known about continuity or change from infancy to mid-childhood. There may be a change in symptom presentation over the course of development and there may not be a gold-standard age to examine anxiety so, to better understand the general presentation of anxiety, assessments should be carried out at different time points.

In contrast, strong early regulation was related to later ASD symptoms and this was only partially mediated by anxiety. Further, the relation between early regulation and later anxiety traits were fully mediated by ASD symptoms. This means that the degree to which early regulation predicts later anxiety is likely accounted for by the fact that children on the path to developing ASD often subsequently develop more symptoms of anxiety. Further, in Model 4 higher EC at 24 months was associated with both reduced anxiety symptoms and ASD traits at 36 months. However, the standardised beta coefficients were over twice as large for the relation between EC and ASD (Fig. 3). Again, this suggests that lower EC is more strongly associated with later ASD than anxiety. One way to interpret this finding is that infants within our cohort with more genetic/early risk factors for ASD have lower levels of EC. This means infants are less likely to compensate if they also happen to be high in BI (due to genetic or other risk factors), leading to a raised risk of subsequently developing anxiety symptoms. So, anxiety may commonly co-occur with ASD because generally lower levels of EC leave children vulnerable to the effects of other risk factors that may vary in the general population.

Model 4 showed a positive association between better EC at 9 months and more ASD symptoms at 36 months. Many of the items that comprise the EC construct at this age ask about how long the child can pay attention to things in their environment. Thus, this significant association may reflect prolonged visual fixations in infants (“sticky attention”;Elsabbagh et al. 2009a, b). This result also corroborates the findings from eye-tracking studies that indicated difficulties in switching attentional focus from peripheral stimuli and longer duration of attention that consistent with processing speed of the exposed stimuli in infants who diagnosed with ASD (Elsabbagh et al. 2009a, b; Richard and Lajiness-O’Neill 2015). From 15 months and on, this association was not significant. Further, by 24 months we saw the expected association between EC at 24 months, and more symptoms of anxiety and ASD at 36 months. By age 2, the EC construct is less heavily influenced by questions about visual attention, engagement, and contains more items asking about inhibitory control, sustained attention. This change in emphasis of the construct may explain this pattern. Alternatively, it may be that atypically strong EC early in development indicates an unusually paced developmental trajectory, which may be a sign of emerging vulnerability. The limited skills of infants may be developmentally beneficial because they facilitate learning (Bjorklund 1997; Elman 1993) and so developing strong EC too early may be a risk factor.

### Limitations and Future Directions

One limitation of the present study was the modest sample size, particularly within the LR group. Due to this, we were unable to examine the multi-group models which would inform us whether such associations are consistent for both HR and the LR groups. Second, the exploratory mediation analyses should be interpreted with caution since some of the mediator and outcome variables (anxiety and ASD) were measured cross-sectionally. Maxwell et al. (2011) stressed that a cross-sectional investigation of mediation might lead to substantial bias. A third limitation arises due to relying on parent-reported data, which may increase the risk of informant bias. Having an older child with an ASD diagnosis might affect the parental judgement for the younger child. Moreover, parental anxiety problems have been found to mediate the relationship between temperament and anxiety problems (Gartstein et al. 2010). This might raise attributional bias and future research should take into account the possible effects of parental psychopathology on childhood anxiety problems. Fourth, another limitation may arise from the instruments used to measure BI. Since the Fear subscales of the IBQ-R includes reactions to novel social and non-social stimuli, it captures unitary constructs of BI well. However, evidence in toddlers and older children indicates that the social and non-social aspects of BI may be separable and differentially related to anxiety (Brooker et al. 2016; Dyson et al. 2011). Indeed, this is why the ECBQ contains separable Fear (non-social) and Shyness (social) subdomains. In the present study, results were consistent if we used the ECBQ Shyness and Fear subdomains as a composite (Supplementary Material Section 7) or the Shyness domain alone (which presented greater developmental stability with infant Fear and a better model fit). Future work should examine whether social and non-social components of infant BI can be better dissociated with multiple methods (e.g., Dyson et al. 2011; Eggum et al. 2009) and whether these social and non-social components are differentially related to later anxiety and ASD from infancy (or whether this only emerges later in development).

Lastly, employing a single method approach may increase the possibility of yielding results that are specific to a single method or due to shared method variance between predictors and outcome. However, our observation that results were specific to BI and not sadness (extracted from the same questionnaire) mitigates this possibility. Further research should employ a multi-method approach encompassing both parent report and observational measurements to boost ecological validity.

## Conclusion

Our study has three key findings. First, early behavioural inhibition predicts later anxiety in infants with older siblings with ASD as is seen in studies conducted in the general population. Second, regulatory control can act as a protective factor on the path to anxiety symptoms. Finally, our findings suggest that anxiety may co-occur with ASD in part because reduced effortful control in ASD reduces the ability to compensate for other background risk factors that then are more likely to lead to psychopathology. Interventions designed to strengthen self-control in late infancy (but perhaps not earlier) could thus be a promising avenue for early intervention approaches.

## Electronic supplementary material

Below is the link to the electronic supplementary material.

Supplementary file1 (DOCX 62 kb)
