# Supplementary material for: Developmental Paths to Anxiety in an Autism-Enriched Infant Cohort: The Role of Temperamental Reactivity and Regulation
Source: J Autism Dev Disord. 2020 Oct 9;51(8):2631–45. doi: 10.1007/s10803-020-04734-7 (PMC8254725; doi:10.1007/s10803-020-04734-7)
Supplement: Supplementary file 1 — Supplementary file1 (DOCX 62 kb) [file 10803_2020_4734_MOESM1_ESM.docx]

# Supplementary Materials for: Developmental Paths to Anxiety in an Autism-Enriched Infant Cohort: The Role of Reactivity and Regulation

## 1. Participants

Of the 116 high-risk (HR) participants, 77 probands met the criteria on both the DAWBA and SCQ, eight probands fell below the SCQ cut-off (≥15) but were included due to meeting the threshold on the DAWBA. For 19 probands, confirmation of ASD diagnosis was only available for the SCQ while for five, neither measure was available, but they were included based on parent-confirmed clinical ASD diagnosis.

### Clinical measures and outcome decision

A battery of clinical measures was used for the outcome group categorisation at 36 months and all participants completed the following measures: The Autism Diagnostic Observation Schedule – Second Edition (ADOS-2; Lord et al., 2012) is a standardised, semi-structured, observational measure that assesses symptoms related to ASD, including communication, social interaction and restricted, repetitive behaviours. The ADOS was not completed for 2 LR children at 36-months, but these participants were included in the analysis. The Calibrated Severity Scores (CSS) for Social Affect and Restricted and Repetitive Behaviours subscales were reported (Gotham, Pickles, & Lord, 2009). The Autism Diagnostic Interview-Revised (ADI-R; Rutter, LeCouteur, et al., 2003) is a structured parent interview consisting of questions about developmental history, and retrospective symptoms and behaviours related to ASD. The ADI-R was administered at 36 months and standard algorithm scores which aggregate current and historical symptom information were computed for Reciprocal Social Interaction, Communication, and Restricted, Repetitive and Stereotyped Behaviours and Interests subdomains. These assessments were carried out by researchers who were not blind to risk group status or under the close supervision of clinical researchers (i.e., psychologists, speech therapists). Children completed the Mullen Scales of Early Learning (MSEL; Mullen, 1995) at each visit and the early learning composite score was used as a measure of overall developmental level.

After the 36-month visit, experienced researchers (TC, GP, CC) reviewed the ADOS-2 and ADI-R for ASD symptomatology and the MSEL for the developmental level of all the HR and LR children to ascertain diagnostic outcome based on DSM-5 criteria for ASD. Subsequently, of 76 HR infants, 29 were typically developing (hereafter, HR-TD), 29 were considered as having neither ASD nor typical development but other atypicalities due to i) scoring above ADI-R cut-off and/or scoring above ADOS-2 cut off for ASD, or ii) scoring 1.5 standard deviation below the general population mean on the MSEL composite or the MSEL expressive or Receptive Language scales (hereafter, HR-ATY), 17 met criteria for ASD diagnosis (hereafter, HR-ASD). All 27 LR children presented typical development and were not given a research or community ASD diagnosis (Table S1).

Table S1

Sample Characteristics of Risk and Outcome Groups

|  | Low-Risk  M (SD) | High-Risk  M (SD) | HR diagnostic outcome groups | | |
| --- | --- | --- | --- | --- | --- |
|  |  |  | Typical | Atypical | ASD |
| **15 months** |  |  |  |  |  |
| N (girls) | 25 (12) | 75 (28) | 29 (15) | 29 (11) | 17 (2) |
| Age in day | 470.40 (4.97) | 439.47(27.92) | 401.47 (68.60) | 467.25 (6.31) | 461.94 (7.10) |
| MSEL ELC | **100.82 (2.83)^a^** | **93.30 (1.79)** | 98.46 (2.35)^a^ | 91.89 (3.19)^b^ | 86.53 (3.28)^b^ |
| **36 months** |  |  |  |  |  |
| N (girls) | 25 (12) | 75 (28) | 29 (15) | 29 (11) | 17 (2) |
| Age in months | 38.74 (.30) | 38.67 (.22) | 38.70 (.31) | 38.67 (.38) | 38.69 (.46) |
| MSEL ELC | **118.13 (3.24)^a^** | **97.35 (3.23)** | 114.46 (3.11)^a^ | 87. 93 (4.98)^b^ | 85.53 (7.50)^b^ |
| ADOS SA CSS | **2.70 (.41)^a^** | **3.42 (.31)** | 1.64 (.16)^a^ | 4.67 (.48)^b^ | 4.33 (.80)^b^ |
| ADOS RRB CSS | **3.26 (.47)^a^** | **4.97 (.30)** | 3.75 (.44)^a^ | 5.33 (.52)^b^ | 6.33 (.42)^b^ |
| ADI Social | **1.00 (.32)^a^** | **4.51 (.66)** | 1.54 (.42)^a^ | 3.30 (.63)^a^ | 12.00 (1.53)^b^ |
| ADI Com | **.52 (.23)^a^** | **5.01 (.62)** | 2.04 (.49)^a^ | 4.48 (.84)^b,c^ | 11.27 (1.23)^b,d^ |
| ADI RRB | **.09 (.06)^a^** | **1.94 (.33)** | .43 (.18)^a^ | 1.44 (.44)^a^ | 5.53 (.68)^b^ |

Bold indicates a significant group difference between LR and overall HR group. Superscript letters indicate a significant difference between the LR and HR outcome groups at *p* <.05 level. MSEL ELC= Mullen Scales of Early Learning Early Learning Composite Scores, ADOS SA CSS= Autism Diagnostic Observational Schedule Social Affect Calibrated Severity Scores, ADOS RRB CSS= Autism Diagnostic Observational Schedule Restricted Repetitive Behaviours Calibrated Severity Scores, ADI Social= Autism Diagnostic Interview Social Subscale, ADI Com= Autism Diagnostic Interview Communication, ADI RRB= Autism Diagnostic Interview Restricted Repetitive Behaviour subscale.

## 2. Pearson Correlation Coefficients of Variables in the HR and the LR Groups Combined

In the main text, Pearson correlation coefficients were calculated for the HR and LR groups separately. We note that the non-significant associations between temperament variables and outcome anxiety and ASD within the LR group may be due to the lack of power. When checking effect sizes, the association between BI 15 months and anxiety was slightly larger in the LR group (LR *r^2^* = .135; HR *r^2^* = .123) and 15 months and anxiety was larger in the LR group (LR *r^2^* = -.158; HR *r^2^* =-.114). Rest of the effect sizes between temperament and anxiety were larger in the HR group (Table 2). Effect size between BI 9 months and ASD (LR *r^2^* = .111; HR *r^2^* =.005); BI 15 months and ASD (LR *r^2^* = .320; HR *r^2^* =.006); Sadness 15 months and ASD (LR *r^2^* = .016; HR *r^2^* =.005); EC 8 months and ASD (LR *r^2^* = .081; HR *r^2^* =.004) was larger in the LR group. Nevertheless, the non-significant associations between the research variables within the LR group allowed us to combine both groups to address the power for the complex models.

In order to show the variation between temperament variables, anxiety and ASD outcome scores prior to running the models, we calculated the Pearson correlation coefficients for the HR and the LR combined sample. The results showed that there are sufficient variations between research variables to run the desired models.

Table S2

Pearson Correlation Coefficients for Associations between Temperament Scales, Anxiety and SCQ Scores in the HR and LR Groups Combined.

|  | 1. | 2. | 3. | 4. | 5. | 6. | 7. | 8. | 9. | 10. | 11. | 12. |
| --- | --- | --- | --- | --- | --- | --- | --- | --- | --- | --- | --- | --- |
| 1. IBQ Fear 9m | ---- |  |  |  |  |  |  |  |  |  |  |  |
| 2. IBQ Fear 15m | .553* | ---- |  |  |  |  |  |  |  |  |  |  |
| 3. ECBQ Shyness 24m | .300 | .520* | ---- |  |  |  |  |  |  |  |  |  |
| 4. ECBQ Fear 24 | .406* | .407* | .590* | ---- |  |  |  |  |  |  |  |  |
| 5. IBQ Sadness 9m | .548* | .287 | .184 | .280 | ---- |  |  |  |  |  |  |  |
| 6. IBQ Sadness 15m | .382* | .392* | .267 | .320* | .560* | ---- |  |  |  |  |  |  |
| 7. ECBQ Sadness 24m | .389* | .474* | .417* | .399* | .451* | .440* | ---- |  |  |  |  |  |
| 8. IBQ Effortful Control 9m | -.111 | -.067 | -.153 | -.109 | -.097 | -.072 | -.101 | ---- |  |  |  |  |
| 9. IBQ Effortful Control 15m | -.052 | -.126 | -.246 | -.172 | -.003 | -.126 | -.134 | .555* | ---- |  |  |  |
| 10. ECBQ Effortful Control 24m | -.105 | -.209 | -.237 | -.180 | -.209 | -.126 | -.247 | .432* | .533* | ---- |  |  |
| 11. CBCL Anxiety Problems 36m | .313* | .369* | .442* | .385* | .253 | .220 | .396* | -.140 | -.345* | -.424* | ---- |  |
| 12. SCQ 36m | .118 | .147 | .312 | .230 | .128 | .095 | .286 | -.041 | -.330* | -.586* | .583* | --- |

* *p* < .001; IBQ: Infant Behaviour Questionnaire, ECBQ: Early Childhood Behaviour Questionnaire, CBCL: Child Behaviour Checklist, SCQ: Social Communication Questionnaire.

The rest of the analyses were run for 143 observed variables using the MLR estimator and the group variable was treated as a covariate.

## 3. Re-running Analysis with the HR and the LR Groups Combined

*Model 1: Longitudinal association between BI and anxiety.*

Fit indices of this model indicated good for the data (*χ^2^* (1) =.27, *p* = .60; CFI = 1.00, RMSEA = .00, and SRMR = .01). The association between the risk group (HR vs LR) and BI at 9 and 15 months was significant (*β* = .15, *p* = .018; *β* = .13, *p* = .043, respectively), indicating higher BI in HR group at these timepoints. The autoregressive paths of BI were significant; BI at 9 months associated with BI at 15 months (*β* = .53, *p* < .001) and BI at 15 months was associated with BI at 24 months (*β* = .52, *p* < .001). As in the main analysis (Model 1 in the main text), only BI at 24 months was related to anxiety at 36 months (*β* = .34, *p* < .001).

*Model 2:* *Specificity of BI in Predicting Anxiety*

The fit indices of the autoregressive model indicated acceptable fit to the data despite significance for the model (*χ^2^* (4) = 11.59, *p* = .021; CFI = .97, RMSEA = .12, and SRMR = .04). Risk group was significantly related to BI at 9 and 15 months and sadness at 24 months (*β* = .15, *p* = .019; *β* = .13, *p* = .040; *β* = .13, *p* = .024; respectively). All coefficients for the autoregressive pathways of BI remained significant; BI at 9 months related to subsequent BI at 15 months (*β* = .54, *p* < .001) and BI at 15 months associated with BI scores at 24 months (*β* = .49, *p* < .001). Sadness at 9 months related to subsequent sadness at 15 months (*β* = .51, *p* < .001) and sadness at 15 months associated with sadness scores at 24 months (*β* = .30, *p* < .001).

As for the cross-lagged paths, higher levels of BI at 15 months related to increased sadness at 24 months (*β* = .32, *p* < .001) and the rest of the cross-lagged associations were not significant (all *p* ≥ .129). There were concurrent associations between BI and sadness at each timepoints (9 months: *β* = .55, *p* < .001; 15 months: *β* = .28, *p* = .002; 24 months: *β* = .22, *p* = .015) but the magnitude of the association decreased over time.

There were no significant relationships between sadness and anxiety problems at any time point (*p* ≥ .133). Greater BI at 24 months was significantly related to higher anxiety scores at 36 months (*β* = .30, *p* = .003). Similar to the analyses in the main text, the predictive relation between BI and anxiety was not shared with sadness.

*Model 3: Longitudinal association between BI, EC and anxiety*

The fit indices of the autoregressive model indicated a good fit to the data (*χ^2^* (4) = 5.36, *p* = .252; CFI = .99, RMSEA = .04, and SRMR = .02). The risk group was significantly related to 9- and 15-months BI and 15 months EC scores (*β* = .15, *p* = .017; *β* = .13, *p* = .045; *β* = -.16, *p* = .010; respectively). All coefficients for the autoregressive pathways of BI remained significant; BI at 9 months related to subsequent BI at 15 months (*β* = .53, *p* < .001) and BI at 15 months associated with BI scores at 24 months (*β* = .50, *p* < .001). Effortful control at 9 months related to subsequent effortful control at 15 months (*β* = .56, *p* < .001) and effortful control at 15 months associated with effortful control scores at 24 months (*β* = .53, *p* < .001).

As for the cross-lagged paths, higher levels of effortful control at 15 months related to decreased BI at 24 months (*β* = -.20, *p* = .009). Higher levels of BI and lower levels of effortful control measured at 24 months were significantly associated with higher levels of anxiety symptoms (*β* = .24, *p* = .008; *β* = -.29, *p* = .014; respectively).

*Model 4: Longitudinal association between BI, EC, anxiety and ASD*

The fit indices of the autoregressive model indicated a good fit to the data (*χ^2^* (4) = 4.20, *p* = .380; CFI = 1.00, RMSEA = .02, and SRMR = .02). Again, the risk group was significantly related to BI at 9 and 15 months, EC at 15 months, and ASD at 36 months (*β* = .15, *p* = .016; *β* = .13, *p* = .043; *β* = -.16, *p* = .010; *β* = .13, *p* = .016; respectively). BI at 9 months related to subsequent BI at 15 months (*β* = .53, *p* < .001) and BI at 15 months associated with BI scores at 24 months (*β* = .50, *p* < .001). Effortful control at 9 months related to subsequent effortful control at 15 months (*β* = .56, *p* < .001) and effortful control at 15 months associated with effortful control scores at 24 months (*β* = .52, *p* < .001). Concurrent correlations between BI and EC were not significant at all three timepoints (*p* ≥.192).

As for the cross-lagged paths, higher levels of effortful control at 15 months related to decreased BI at 24 months (*β* = -.20, *p* = .010). Effortful control at 9 months was associated with ASD at 36 months (*β* = .28, *p* < .001). Both higher levels of BI and lower levels of effortful control at 24 months were significantly associated with higher levels of anxiety symptoms (*β* = .24, *p* = .007; *β* = -.28, *p* = .013; respectively) and ASD symptoms (*β* = .20, *p* = .003; *β* = -.61, *p* < .001; respectively). Anxiety and ASD scores were significantly associated with each other at 36 months (*β* = .43, *p* < .001). Similar to the analysis with the HR only group, results indicate that the combination of higher EC capacity and lower BI may be a risk of anxiety and ASD.

## Exploratory Mediation Analysis Run on the Combined HR and LR Group

In the main text, we run the exploratory mediation analysis only in the HR group, to examine whether power influence the results, we re-run the same mediation analyses on the combined HR and the LR. There was an indirect effect of BI (24 months) on anxiety (36 months) via ASD (36 months; *β =* .14, *p* = .004, 95% CI [.06, .22]). The direct (*β* = -.28, SE = .08, *p* = .001), and the total effects *(β* = .42, SE = .10, *p* < .001) were significant, suggesting a partial mediation. Specifically, 33% of the total effect of BI on anxiety was operating through ASD. There was also a significant indirect effect of BI on ASD through anxiety (*β* = .22, *p* < .001, 95% CI [.12, .32]). The direct effect of BI on ASD was not significant *(β* = .06, SE = .09, *p =* .472), and the total effect was significant *(β* = .28, SE = .10, *p =* .004), anxiety mediated (complete mediation) the relationship between BI and ASD and accounted for 79% of the total effect. Thus, these results suggest that BI is more strongly related to anxiety rather than ASD traits.

The indirect effect of EC (24 months) on anxiety (36 months) via ASD (36 months) was also significant (*β = -*.31, *p* < .001, 95% CI [-.41, -.21]). The direct effect was not significant (*β* = -.09, SE = .09, *p* = .331), and the total effect was significant *(β* = -.39, SE = .08, *p* < .001), suggesting a complete mediation. Seventy-nine per cent of the total effect of EC on anxiety was operating through ASD. There was also a significant indirect effect of EC on ASD through anxiety (*β* = -.16, *p* = .001, 95% CI [-.25, -.08]). The direct effect *(β* = -.43, SE = .09, *p <* .001), and the total effect were both significant *(β* = -.59, SE = .07, *p* < .01). Anxiety partially mediated the association between EC and ASD, accounting for the 27% of the total effect. Thus, these results support the results from the HR only mediation analysis in the main text.

## 4. Re-running Analysis with Listwise Deletion

Models 1-4 were re-calculated on 89 participants, omitting the participants that have not got the all variables.

*Model 1: Longitudinal association between BI and anxiety.*

The fit indices of the autoregressive model indicated a good fit to the data (*χ^2^* (1) =.007, *p* = .931; CFI = 1.00, RMSEA = .000, and SRMR = .002). All coefficients for the autoregressive pathways of BI remained significant; BI at 9 months related to subsequent BI at 15 months (*β* = .56, *p* < .001) and BI at 15 months associated with BI scores at 24 months (*β* = .54, *p* < .001), indicating stability between time points. Regarding the timing, higher levels of BI at 24 months were associated with higher levels of anxiety at 36 months (*β* = .42, *p* < .001). All other associations between the temperament variables and anxiety scores were not significant (all *p* ≥ .489).

*Model 2:* *Specificity of BI in Predicting Anxiety*

The fit indices of the autoregressive model indicated acceptable fit to the data (*χ^2^* (4) = 9.945, *p* = .041; CFI = .965, RMSEA = .129, and SRMR = .038). All coefficients for the autoregressive pathways of BI remained significant; BI at 9 months related to subsequent BI at 15 months (*β* = .58, *p* < .001) and BI at 15 months associated with BI scores at 24 months (*β* = .53, *p* < .001). Sadness at 9 months related to subsequent sadness at 15 months (*β* = .53, *p* < .001) and sadness at 15 months associated with sadness scores at 24 months (*β* = .27, *p* < .001).

As for the cross-lagged paths, higher levels of BI at 15 months related to increased sadness at 24 months (*β* = .37, *p* < .001) and the rest of the cross-lagged associations were not significant (all *p* ≥ .767). There were concurrent associations between BI and sadness at each timepoints (9 months: *β* = .60, *p* < .001; 15 months: *β* = .36, *p* < .001; 24 months: *β* = .25, *p* = .013) but the magnitude of the relationship decreased over time.

There were no significant relationships between sadness and anxiety problems at any time point (all *p* ≥ .056). Greater BI at 24 months was significantly related to higher anxiety scores at 36 months (*β* = .39, *p* < .001).

*Model* *3: Longitudinal association between BI, EC and anxiety*

The fit indices of the autoregressive model indicated a good fit to the data (*χ^2^* (4) = 2.44, *p* = .654; CFI = 1.00, RMSEA = .000, and SRMR = .020). All coefficients for the autoregressive pathways of BI remained significant; BI at 9 months related to subsequent BI at 15 months (*β* = .56, *p* < .001) and BI at 15 months associated with BI scores at 24 months (*β* = .52, *p* < .001). Effortful control at 9 months related to subsequent effortful control at 15 months (*β* = .58, *p* < .001) and effortful control at 15 months associated with effortful control scores at 24 months (*β* = .53, *p* < .001).

As for the cross-lagged paths, higher levels of effortful control at 15 months related to decreased BI at 24 months (*β* = -.24, *p* = .003). All other cross-lagged paths were not significant (*p* ≥ .164). Both higher levels of BI and lower levels of effortful control at 24 months were significantly associated with higher levels of anxiety symptoms (*β* = .31, *p* = .003; *β* = -.29, *p* = .027; respectively).

*Model 4: Longitudinal association between BI, EC, anxiety and ASD*

The fit indices of the autoregressive model indicated a good fit to the data (*χ^2^* (4) = 2.444, *p* = .654; CFI = 1.00, RMSEA = .000, and SRMR = .021). All coefficients for the autoregressive pathways of BI remained significant; BI at 9 months related to subsequent BI at 15 months (*β* = .56, *p* < .001) and BI at 15 months associated with BI scores at 24 months (*β* = .52, *p* < .001). Effortful control at 9 months related to subsequent effortful control at 15 months (*β* = .58, *p* < .001) and effortful control at 15 months associated with effortful control scores at 24 months (*β* = .53, *p* < .001).

As for the cross-lagged paths, higher levels of effortful control at 15 months related to decreased BI at 24 months (*β* = -.24, *p* = .006). All other cross-lagged paths were not significant (all *p* ≥ .149). Effortful control at 9 months was associated with ASD at 36 months (*β* = .26, *p* = .006). Both higher levels of BI and lower levels of effortful control at 24 months were significantly associated with higher levels of anxiety symptoms (*β* = .31, *p* = .003; *β* = -.29, *p* = .005; respectively) and ASD symptoms (*β* = .25, *p* = .010; *β* = -.61, *p* < .001; respectively). Anxiety and ASD was significantly associated with each other at 36 months (*β* = .44, *p* < .001).

## 5. Re-Running Analysis Covarying for Sex

*Model 1: Longitudinal association between BI and anxiety.*

The fit indices of the autoregressive model indicated a good fit to the data (*χ^2^* (1) = .031, *p* = .859; CFI = 1.00, RMSEA = .00, and SRMR = .003). Sex was not significantly related to any variables (*p* ≥ .059). All coefficients for the autoregressive pathways of BI remained significant; BI at 9 months related to subsequent BI at 15 months (*β* = .52, *p* < .001) and BI at 15 months associated with BI scores at 24 months (*β* = .50, *p* < .001), indicating stability between time points. Regarding the timing, higher levels of BI at 24 months were associated with higher levels of anxiety at 36 months (*β* = .37, *p* < .001).

*Model 2:* *Specificity of BI in Predicting Anxiety*

Overall model fit for the second model was acceptable (CFI = .97, RMSEA = .12, and SRMR = .03), despite significance for the model (*χ^2^* (4) = 12.65, *p* = .013). Sex was not significantly associated with any variables (all *p* ≥ .065). The autoregressive pathways indicated significant associations for BI variables between 9 and15 months: *β* = .52, *p* < .001, 15 and 24 months (*β* = .49, *p* < .001); and for sadness between 9 and 15 months (*β* = .53, *p* < .001), 15 and 24 months (*β* = .29, *p* = .002). BI and sadness was associated with each other concurrently at each time point (9 months: *β* = .56, *p* < .001; 15 months: *β* = .32, *p* = .001; 24 months: *β* = .23, *p* = .021) but the magnitude of the relationship decreased over time.

Cross-lagged paths indicated that more BI at 15 months was related to more sadness at 24 months (*β* = .31, *p* = .003) and more sadness at 15 months was related to more BI at 24 months (*β* = .31, *p* = .003). The rest of the cross-lagged paths were not significant (all *p* ≥ .530). Greater BI at 24 months was significantly related to higher anxiety scores at 36 months (*β* = .33, *p* = .002) and the all the relationships between other temperament variables and anxiety problems were not significant (all *p* ≥ .084).

*Model 3: Longitudinal association between BI, EC and anxiety*

The fit indices of the autoregressive model indicated a good fit to the data (*χ^2^* (4) = 4.20, *p* = .380; CFI = .999, RMSEA = .021, and SRMR = .026). Sex was significantly related to effortful control at 24 months (*β* = .22, *p* = .005) and the rest of the associations were not significant (all *p* ≥ .059). All coefficients for the autoregressive pathways of BI remained significant; BI at 9 months related to subsequent BI at 15 months (*β* = .51, *p* < .001) and BI at 15 months associated with BI scores at 24 months (*β* = .47, *p* < .001). Effortful control at 9 months related to subsequent effortful control at 15 months (*β* = .55, *p* < .001) and effortful control at 15 months associated with effortful control scores at 24 months (*β* = .51, *p* < .001).

As for the cross-lagged paths, higher levels of effortful control at 15 months related to decreased BI at 24 months (*β* = -.20, *p* = .014) and the rest of the cross-lagged associations were not significant (all *p* ≥ .608). Both higher levels of BI and lower levels of effortful control at 24 months were significantly associated with higher levels of anxiety symptoms (*β* = .26, *p* = .006; *β* = -.30, *p* = .019; respectively). All other associations between temperament variables and anxiety were not significant (all *p* ≥ .053).

*Model 4:* *Longitudinal association between BI, EC, anxiety and ASD*

The fit indices of the autoregressive model indicated a good fit to the data (*χ^2^* (4) = 3.02, *p* = .555; CFI = 1.00, RMSEA = .000, and SRMR = .021). Sex was significantly related to effortful control at 24 months (*β* = .24, *p* = .003) and all other associations between sex and variables were not significant (all *p* ≥ .063). All coefficients for the autoregressive pathways of BI remained significant; BI at 9 months related to subsequent BI at 15 months (*β* = .51, *p* < .001) and BI at 15 months associated with BI scores at 24 months (*β* = .47, *p* < .001). Effortful control at 9 months related to subsequent effortful control at 15 months (*β* = .55, *p* < .001) and effortful control at 15 months associated with effortful control scores at 24 months (*β* = .51, *p* < .001).

As for the cross-lagged paths, higher levels of effortful control at 15 months related to decreased BI at 24 months (*β* = -.20, *p* = .016) and the rest of the cross-lagged associations were not significant (all *p* ≥ .057). Effortful control at 9 months was associated with ASD at 36 months (*β* = .27, *p* = .002). There were negative associations between BI at 15 months and ASD (*β* = -.20, *p* = .035). Both higher levels of BI and lower levels of effortful control at 24 months were significantly associated with higher levels of anxiety symptoms (*β* = .27, *p* = .007; *β* = -.29, *p* = .006; respectively) and ASD symptoms (*β* = .23, *p* = .008; *β* = -.63, *p* < .001; respectively).

## 6. Re-Running Analysis Covarying for the Mullen Early Learning Composite (MSEL) Score

The Mullen Scales of Early Learning (MSEL; Mullen, 1995) is a performance-based developmental measure and consisting of four subscales: visual reception, fine motor, receptive and expressive language that are combined to calculate an early learning composite score. The composite MSEL score that was measured at 36 months visit was covaried in the analyses and regressed on only anxiety and ASD scores to control for the effect of the IQ.

*Model 1: Longitudinal association between BI and anxiety.*

The fit indices of the autoregressive model indicated a good fit to the data (*χ^2^* (1) = .041, *p* = .838; CFI = 1.00, RMSEA = .00, and SRMR = .004). The MSEL scores was negatively related to anxiety at 36 months (*β* = -.22, *p* = .009), the associations between temperament variables and MSEL scores were not significant (all *p* ≥ .110). All coefficients for the autoregressive pathways of BI remained significant; BI at 9 months related to subsequent BI at 15 months (*β* = .54, *p* < .001) and BI at 15 months associated with BI scores at 24 months (*β* = .51, *p* < .001), indicating stability between time points. Regarding the timing, higher levels of BI at 24 months were associated with higher levels of anxiety at 36 months (*β* = .36, *p* < .001) and the association between 9 months BI and anxiety was significant (*β* = .22, *p* = .023).

*Model 2:* *Specificity of BI in Predicting Anxiety*

The fit indices of the autoregressive model indicated an acceptable fit to the data (*χ^2^* (4) = 12.17, *p* = .016; CFI = .963, RMSEA = .136, and SRMR = .037). There were not significant associations between temperament variables and the MSEL scores at any time point (all *p* ≥ .139) and there was a negative association between the MSEL scores and anxiety (*β* = -.20, *p* = .011).

The autoregressive pathways indicated significant associations for BI variables between 9 and15 months: *β* = .55, *p* < .001, 15 and 24 months (*β* = .51, *p* < .001); and for sadness between 9 and 15 months (*β* = .54, *p* < .001), 15 and 24 months (*β* = .32, *p* < .001). BI and sadness was associated with each other concurrently at each time point (9 months: *β* = .57, *p* < .001; 15 months: *β* = .34, *p* = .001; 24 months: *β* = .29, *p* = .002) but the magnitude of the relationship decreased over time.

Cross-lagged paths indicated that more BI at 15 months was related to more sadness at 24 months (*β* = .34, *p* = .001) but there were no significant relationships between sadness and anxiety problems at any time point (*p* ≥ .187). Greater BI at 24 months was significantly related to higher anxiety scores at 36 months (*β* = .30, *p* = .002) and rest of the associations between temperament variables and anxiety were not significant (all *p* ≥ .157).

*Model 3: Longitudinal association between BI, EC and anxiety*

The fit indices of the autoregressive model indicated a good fit to the data (*χ^2^* (4) = 4.42, *p* = .352; CFI = .997, RMSEA = .031, and SRMR = .026). The MSEL scores was significantly related to anxiety at 36 months (*β* = -.16, *p* = .026). There were negative association between MSEL and 9 months effortful control scores (*β* = .23, *p* = .009) and rest of the associations between temperament variables and MSEL were not significant (all *p* ≥ .056).

All coefficients for the autoregressive pathways of BI remained significant; BI at 9 months related to subsequent BI at 15 months (*β* = .53, *p* < .001) and BI at 15 months associated with BI scores at 24 months (*β* = .49, *p* < .001). Effortful control at 9 months related to subsequent effortful control at 15 months (*β* = .54, *p* < .001) and effortful control at 15 months associated with effortful control scores at 24 months (*β* = .55, *p* < .001).

As for the cross-lagged paths, association between effortful control at 15 months and BI at 24 months become non-significant (*β* = -.16, *p* = .010). BI at 9 months was significantly related to anxiety (*β* = .27, *p* = .018). Both higher levels of BI and lower levels of effortful control at 24 months were significantly associated with higher levels of anxiety (*β* = .27, *p* = .005; *β* = -.23, *p* = .042; respectively) and rest of the associations between temperament variables and anxiety scores were not significant (all *p* ≥ .154).

*Model 4: Longitudinal association between BI, EC, anxiety and ASD*

The fit indices of the autoregressive model indicated an good fit to the data (*χ^2^* (4) = 3.49, *p* = .480; CFI = 1.00, RMSEA = .00, and SRMR = .023). The MSEL score was related to only anxiety at 36 months (*β* = -.15, *p* = .036) and rest of the associations between temperament variables and MSEL were not significant (all *p* ≥ .076).

All coefficients for the autoregressive pathways of BI remained significant; BI at 9 months related to subsequent BI at 15 months (*β* = .53, *p* < .001) and BI at 15 months associated with BI scores at 24 months (*β* = .49, *p* < .001). Effortful control at 9 months related to subsequent effortful control at 15 months (*β* = .54, *p* < .001) and effortful control at 15 months associated with effortful control scores at 24 months (*β* = .54, *p* < .001). As for the cross-lagged paths, unlike reported in the main text, effortful control at 15 months was not related to BI at 24 months after controlling for the MSEL scores (*β* = -.16, *p* = .060) and also rest of the cross-lagged associations were not significant (all *p* ≥ .060).

BI at 15 months negatively associated with the ASD scores (*β* = -.22, *p* = .022); effortful control at 9 months was related to ASD at 36 months (*β* = .23, *p* = .006). There was also a significant association between BI at 9 months and anxiety (*β* = .21, *p* = .031). Both higher levels of BI and lower levels of effortful control at 24 months were significantly associated with higher levels of anxiety (*β* = .27, *p* = .006; *β* = -.23, *p* = .031; respectively) and ASD scores (*β* = .24, *p* = .006; *β* = -.59, *p* < .001; respectively). Anxiety and ASD scores were significantly related to each other (*β* = .42, *p* < .001)*.*

## 7. Re-Running Analysis with Combined Fear and Shyness subscales of ECBQ at 24 Months

*Model 1: Longitudinal association between BI and anxiety.*

The fit indices of the autoregressive model indicated an acceptable fit to the data (*χ^2^* (1) = 2.38, *p* = .122; CFI = .983, RMSEA = .111, and SRMR = .035). All coefficients for the autoregressive pathways of BI remained significant; BI at 9 months related to subsequent BI at 15 months (*β* = .53, *p* < .001) and BI at 15 months associated with BI scores at 24 months (*β* = .55, *p* < .001), indicating stability between time points. Regarding the timing, higher levels of BI at 24 months were associated with higher levels of anxiety at 36 months (*β* = .41, *p* < .001) and the rest of the associations were not significant (all *p* ≥ .260).

*Model 2:* *Specificity of BI in Predicting Anxiety*

The fit indices of the autoregressive model indicated an acceptable fit to the data (*χ^2^* (4) = 14.75, *p* = .005; CFI = .938, RMSEA = .152, and SRMR = .044). The autoregressive pathways indicated significant associations for BI variables between 9 and15 months: *β* = .54, *p* < .001, 15 and 24 months (*β* = .51, *p* < .001); and for sadness between 9 and 15 months (*β* = .52, *p* < .001), 15 and 24 months (*β* = .29, *p* = .002). BI and sadness were associated with each other concurrently at each time point (9 months: *β* = .57, *p* < .001; 15 months: *β* = .32, *p* = .001; 24 months: *β* = .29, *p* = .001) but the magnitude of the relationship decreased over time.

Cross-lagged paths indicated that more BI at 15 months was related to more sadness at 24 months (*β* = .33, *p* = .001) but there were no significant relationships between sadness and anxiety problems at any time point (*p* ≥ .271). Greater BI at 24 months was significantly related to higher anxiety scores at 36 months (*β* = .37, *p* = .002). All other associations between temperament variables and anxiety scores were not significant (all *p* ≥ .182).

*Model 3: Longitudinal association between BI, EC and anxiety*

The fit indices of the autoregressive model indicated an acceptable fit to the data (*χ^2^* (4) = 5.783, *p* = .216; CFI = .989, RMSEA = .062, and SRMR = .033).

All coefficients for the autoregressive pathways of BI remained significant; BI at 9 months related to subsequent BI at 15 months (*β* = .53, *p* < .001) and BI at 15 months associated with BI scores at 24 months (*β* = .53, *p* < .001). Effortful control at 9 months related to subsequent effortful control at 15 months (*β* = .55, *p* < .001) and effortful control at 15 months associated with effortful control scores at 24 months (*β* = .55, *p* < .001).

As for the cross-lagged paths, higher levels of effortful control at 15 months related to decreased BI at 24 months (*β* = -.17, *p* = .014). All other cross-lagged paths were not significant (all *p* ≥ .304). Again, both higher levels of BI and lower levels of effortful control at 24 months were significantly associated with higher levels of anxiety (*β* = .30, *p* = .002; *β* = -.30, *p* = .018; respectively). All other associations between temperament variables and anxiety were not significant (all *p* ≥ .149).

*Model 4: Longitudinal association between BI, EC, anxiety and ASD*

The fit indices of the autoregressive model indicated an good fit to the data (*χ^2^* (4) = 4.757, *p* = .313; CFI = .997, RMSEA = .040, and SRMR = .028). All coefficients for the autoregressive pathways of BI remained significant; BI at 9 months related to subsequent BI at 15 months (*β* = .53, *p* < .001) and BI at 15 months associated with BI scores at 24 months (*β* = .54, *p* < .001). Effortful control at 9 months related to subsequent effortful control at 15 months (*β* = .55, *p* < .001) and effortful control at 15 months associated with effortful control scores at 24 months (*β* = .55, *p* < .001). As for the cross-lagged paths, higher levels of effortful control at 15 months related to decreased BI at 24 months (*β* = -.17, *p* = .030).

Effortful control at 9 months was related to ASD at 36 months (*β* = .26, *p* = .002). Both higher levels of BI and lower levels of effortful control at 24 months were significantly associated with higher levels of anxiety (*β* = .31, *p* = .003; *β* = -.28, *p* = .005; respectively) and ASD symptoms (*β* = .23, *p* = .012; *β* = -.62, *p* < .001; respectively). Anxiety and ASD scores were significantly related to each other (*β* = .43, *p* < .001)*.*

## 8. Baseline Model (Model 1) with Fear Subscale at 24 Months

The fit indices of the autoregressive model indicated a poorer fit to the data specifically, due to inflated RMSEA (*χ^2^* (1) = 5.482, *p* = .019; CFI = .936, RMSEA = .197, and SRMR = .056). As for the autoregressive pathways, fear at 9 months related to subsequent fear at 15 months (*β* = .53, *p* < .001) and fear at 15 months associated with fear scores at 24 months (*β* = .48, *p* < .001). Regarding the timing, higher levels of fear at 24 months were associated with higher levels of anxiety at 36 months (*β* = .29, *p* = .015).
